# An Automated Text Messaging Intervention to Reduce Substance Use Self-Stigma (Project RESTART): Protocol for a Feasibility and Acceptability Pilot Study

**DOI:** 10.2196/59224

**Published:** 2024-08-09

**Authors:** Adams L Sibley, Seth M Noar, Kathryn E Muessig, Nisha G O'Shea, Catherine E Paquette, Abby G Spears, William C Miller, Vivian F Go

**Affiliations:** 1 Department of Health Behavior University of North Carolina-Chapel Hill Chapel Hill, NC United States; 2 Hussman School of Journalism and Media University of North Carolina-Chapel Hill Chapel Hill, NC United States; 3 Institute on Digital Health and Innovation College of Nursing Florida State University Tallahassee, FL United States; 4 Research Triangle Institute Research Triangle Park, NC United States; 5 Department of Population Health Sciences Duke University Durham, NC United States; 6 Scioto Connect Portsmouth, OH United States; 7 Department of Epidemiology University of North Carolina-Chapel Hill Chapel Hill, NC United States

**Keywords:** substance use, harm reduction, stigma, self-stigma, stigma resistance, mobile health, mHealth, SMS text messaging, mobile phone

## Abstract

**Background:**

Stigma is a barrier to treatment and harm reduction seeking in people who use drugs. Most stigma reduction interventions offer psychotherapy or psychoeducation in group-based clinical settings, failing to reach people who are not in treatment. SMS text messaging is an effective and acceptable modality for delivering health information to people who use drugs and may be a suitable conduit for providing information and advice to understand and cope with stigma.

**Objective:**

This paper presents the protocol for a study that aims to determine the feasibility, acceptability, and preliminary effectiveness of a 4-week automated SMS text message intervention to increase stigma resistance and reduce self-stigma in people who use drugs.

**Methods:**

We designed a novel automated SMS text message intervention to address the four personal-level constructs of stigma resistance: (1) not believing stigma and catching and challenging stigmatizing thoughts, (2) empowering oneself through learning about substance use and one’s recovery, (3) maintaining one’s recovery and proving stigma wrong, and (4) developing a meaningful identity and purpose apart from one’s substance use. Theory-based messages were developed and pilot-tested in qualitative elicitation interviews with 22 people who use drugs, resulting in a library of 56 messages. In a single-group, within-subjects, community-based pilot trial, we will enroll 30 participants in the Resisting Stigma and Revaluating Your Thoughts (RESTART) intervention. Participants will receive 2 daily SMS text messages for 4 weeks. Implementation feasibility will be assessed through recruitment, enrollment, retention, and message delivery statistics. User feasibility and acceptability will be assessed at follow-up using 23 survey items informed by the Theoretical Framework of Acceptability. Primary effectiveness outcomes are changes in self-stigma (Substance Abuse Self-Stigma Scale) and stigma resistance (Stigma Resistance Scale) from baseline to follow-up measured via a self-administered survey. Secondary outcomes are changes in hope (Adult Dispositional Hope Scale) and self-esteem (Rosenberg Self-Esteem Scale). Feasibility and acceptability will be assessed with descriptive statistics; effectiveness outcomes will be assessed with paired 2-tailed *t* tests, and group differences will be explored using ANOVA. Overall, 12 participants will also be selected to complete acceptability interviews.

**Results:**

This pilot study was funded by the National Institute on Drug Abuse in April 2023 and received regulatory approval in January 2024 by the University of North Carolina-Chapel Hill Institutional Review Board. Recruitment and enrollment began in March 2024. Follow-up visits are expected to conclude by May 2024. Results will be disseminated in relevant peer-reviewed journals.

**Conclusions:**

To the best of our knowledge, this is the first study to address substance use stigma via a self-help SMS text messaging program. Results will add to the nascent literature on stigma reduction in people who use drugs. This protocol may interest researchers who are considering text messaging to address psychosocial needs in hard-to-reach populations.

**Trial Registration:**

ClinicalTrials.gov NCT06281548; https://clinicaltrials.gov/ct2/show/NCT06281548

**International Registered Report Identifier (IRRID):**

DERR1-10.2196/59224

## Introduction

### Background

Nonmedical use of controlled substances is a leading cause of morbidity and mortality in the United States, with 107,941 reported overdose deaths in 2022 and increasing rates of hepatitis C, endocarditis, and other infectious diseases in people (or person) who use drugs (PWUD) [1-3]. Although evidence-based treatment and harm reduction services are associated with reduced burden of disease [4-10], they remain underused; in the United States, only 6.5% of people with substance use disorders received past-year treatment in 2021 [11]. The role of stigma in the epidemic has come increasingly into focus among policy makers and interventionists [12-16]. Stigma—the social devaluation of PWUD manifesting in stereotypes, prejudice, and discrimination—inhibits help-seeking, promotes substance use as a coping mechanism, and increases the risk of overdose [17-20].

The stigma reduction agenda has, to date, focused overwhelmingly on public stigma—a notoriously slow and challenging target for change—with less research on equipping PWUD to understand and cope with stigmatizing thoughts and experiences [21,22]. Proponents of empowerment approaches (the *self-worth agenda*) suggest that groups considered marginalized should be given resources to understand and challenge their marginalization [13,23,24]. Self-stigma is a salient consequence of public stigma in PWUD. Self-stigma includes self-devaluation (ie, internalized stigma), the fear of encountering public stigma, avoidance of difficult thoughts and experiences, and disengagement from pursuing valued life goals [25]. Self-stigma mediates the association between public stigma and numerous health outcomes, including overdose, making it an important target for intervention [26-31].

A recent systematic review identified 15 intervention trials between 2011 and 2023 with promising evidence of effectiveness in reducing substance use self-stigma [32]. However, almost all studies were in clinical treatment facilities and group settings, ironically failing to reach PWUD who may avoid these settings because of fear of stigma [32].

Noting these gaps, we developed a novel self-help stigma reduction intervention for PWUD in active use premised on the theory of stigma resistance [33]. We chose SMS text messaging as the delivery modality. SMS text message interventions are effective and cost-effective vehicles for addressing substance use and mental health disorders, and PWUD have deemed SMS text messaging an acceptable way to receive health information [34-42]. SMS text messaging also removes some structural barriers (eg, transportation) that would otherwise preclude many PWUD from participating [43].

### Objectives

In this paper, we describe the rationale, theoretical framework, and development of a 4-week, automated SMS text message intervention to increase stigma resistance and reduce self-stigma in PWUD. We then describe the protocol for a single-group pilot trial to determine the feasibility, acceptability, and preliminary effectiveness of the intervention. We hypothesize that the intervention will be feasible from the perspective of implementers and PWUD participants; that participants will find the intervention acceptable per our 16-item Theoretical Framework of Acceptability questionnaire; and that participants will experience reductions in self-stigma and improvements in stigma resistance, hope, and self-esteem from baseline to follow-up.

## Methods

### Ethical Considerations

The protocol was approved by the University of North Carolina-Chapel Hill (UNC) Institutional Review Board in January 2024 (23-2937) and aligns with the principles of the Helsinki Declaration of 1975 (as revised in 2000). Participants will provide written informed consent before beginning any study activities. The informed consent procedure follows the UNC-required consent template, including describing the purpose of the study, procedures to be followed, and risks and benefits of participation. The consent forms use language that is sufficiently simple for laypersons to comprehend. Study staff will be trained to probe for comprehension and to ensure participants understand the voluntariness of their participation. Study records will be stored on password-protected UNC servers; only Institutional Review Board–approved study members will have access. Names are only stored on signed consent forms and will be stored in locked cabinets separately from the data. No identifying data will be published in any reports. Total compensation is up to US $75 in gift cards (US $30 for each survey and US $15 for the follow-up interview).

### Intervention Overview

**Figure 1:**
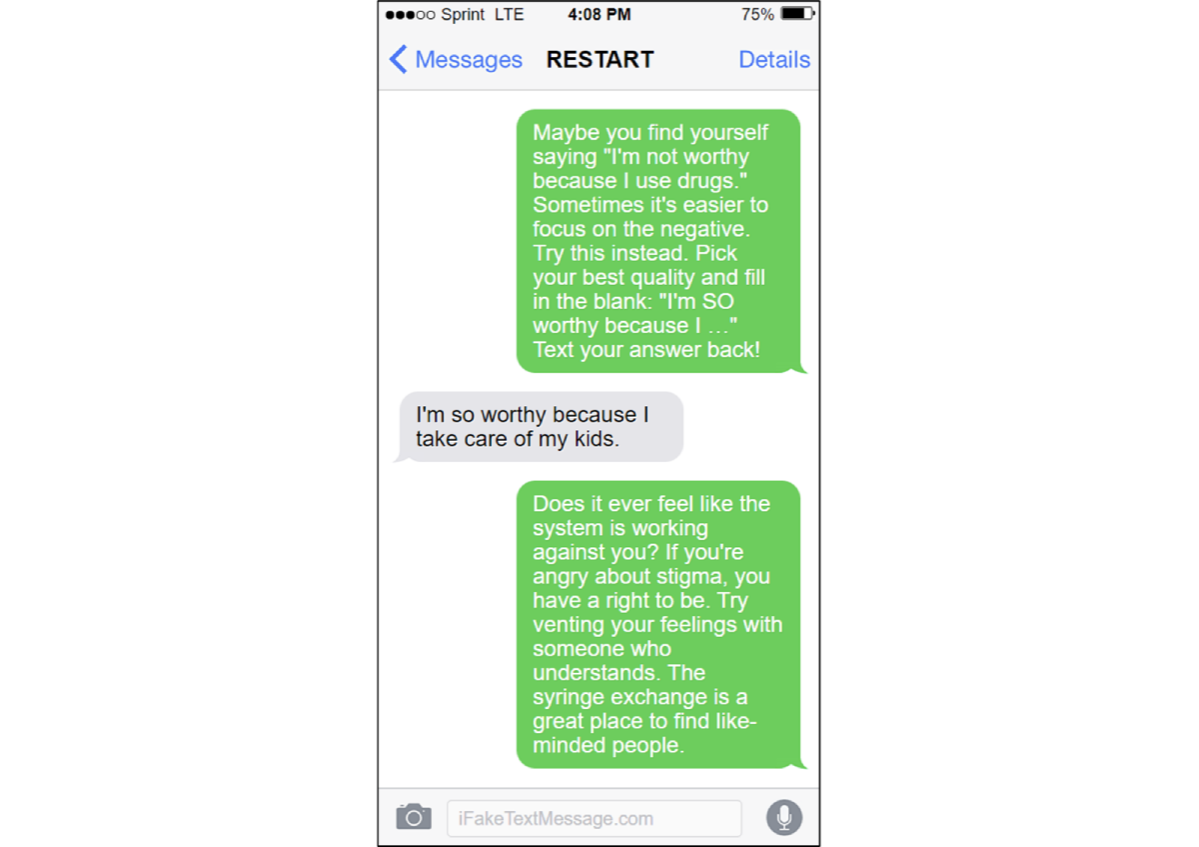
Mock-up of the Resisting Stigma and Revaluating Your Thoughts (RESTART) text messages.

Resisting Stigma and Revaluating Your Thoughts (RESTART) is a theory-informed, 4-week automated SMS text message intervention to reduce self-stigma in PWUD (Figure 1). The general approach of RESTART is to equip PWUD with knowledge and skills to understand, cope with, and ultimately resist substance-related stigma. The intervention delivers 2 daily SMS text messages to participants for 4 weeks (56 messages total).

### Theoretical Framework

#### Stigma Resistance

RESTART promotes stigma resistance, a psychosocial process associated with reduced self-stigma and improvements in self-efficacy, hope, self-esteem, mental health, and quality of life [44-48]. Stigma resistance is the active “opposition to the imposition of mental illness stereotypes by others”; high resisters can challenge or deflect stigmatizing attitudes and behaviors and engage in problem-centered rather than avoidant coping styles [46,49,50]. Firmin et al [33] conceptualized stigma resistance strategies at 3 levels: personal (ie, addressing one’s stigmatizing thoughts and behaviors), peer (ie, helping others to resist stigma), and public (ie, educating, advocating, and publicly challenging stigma).

RESTART addresses the four components of the personal level of stigma resistance [33]: (1) not believing stigma and catching and challenging stigmatizing thoughts, (2) empowering oneself through learning about substance use and one’s recovery, (3) maintaining one’s recovery and proving stigma wrong, and (4) developing a meaningful identity and purpose apart from one’s substance use. We determined that addressing the peer and public levels of stigma resistance (eg, promoting formal peer service involvement and advocacy) would be infeasible in a short-term messaging intervention, as these would likely require more intensive training and nuanced discussions on disclosure decisions.

Although the constructs of stigma resistance provide a suitable target for change, the theory does not provide guidance on *how* to change attitudes, beliefs, or behaviors [51]. Effective health communication interventions integrate theory from 3 domains: behavior change (which attitudes or beliefs are targeted), information processing (how attention, engagement, and acceptance of messages can be maximized), and message effects (format and content of messages expected to be persuasive and promote change; Table 1) [52].

**Table 1:** Theoretical framework for Resisting Stigma and Revaluating Your Thoughts (RESTART).

Theory domain	Applied theories	Role in intervention
Behavior change	Stigma resistance and self-stigma	*Which* attitudes and beliefs are targeted for change?
Information processing	Elaboration likelihood model	*What* mechanisms are needed to promote effortful engagement with the messages?
Message effects	Cognitive restructuring, metacognitive awareness, psychoeducation, cognitive defusion, exemplification, emotional appeals, tailoring, etc	*How* can messages be designed (format or content) to persuade desired cognitive, attitudinal, and emotional outcomes?

#### Information Processing

Intervention development was informed by the elaboration likelihood model, an information processing theory that is helpful for understanding how individuals engage with persuasive messages and how engagement can lead to enduring attitude change [53]. For messages to influence attitude and behavior change, per the persuasion communication model by McGuire [54], recipients must pay attention to the message, comprehend its meaning, evaluate its content, agree with the message, and store its content in memory for later retrieval [52]. Attributes of the message’s source, recipient, context, and content will influence the level of effortful processing (ie, elaboration) by recipients and, ultimately, the message’s impact on sustained attitude and behavior change [53,55]. Motivation and the ability to attend to messages can encourage effortful processing [53,56,57]. Examples of strategies informed by the elaboration likelihood model are included in Table 2.

**Table 2:** Example persuasion strategies informed by the elaboration likelihood model.

Elaboration determinant	Strategy	Activity
Motivation	Increase personal relevance	Reference stereotypes or stigmatizing beliefs that resonate with participants
Ability	Minimize distractions	Send messages at the time of day when participants are least distracted (eg, not at work or driving)
Ability	Use repetition	Reiterate messages in subsequent delivery times or days using similar content but different wording

#### Message Effects

Message effects were drawn from both the psychotherapeutic and health communication literatures. In a recent systematic review of substance use self-stigma interventions, acceptance and commitment therapy, cognitive behavioral therapy (CBT), and psychoeducation were the most common and efficacious approaches to address self-stigma in clinical encounters [32]. We reviewed acceptance and commitment therapy, CBT, and psychoeducational manuals to identify modalities that could be translated into brief messages. For instance, cognitive restructuring, a component of CBT, suggests identifying and challenging one’s distorted thought patterns (such as overgeneralizing or catastrophizing) and reframing these thoughts in more healthy and productive ways [58]. A message using this approach might read as follows:

Have you had a bad thought about yourself this week? Let’s think of a reason that thought is NOT true. Here’s an example: “My family might think I’m selfish. But that’s not necessarily true. To be the best I can be, I have to take care of myself first.”

We incorporated multiple approaches from the health communication literature, including exemplification and tailoring, into our messages. Exemplification involves using specific exemplars (such as a celebrity in recovery) that are iconic, relatable, and emotionally arousing instead of generic representations [59]. Tailoring involves designing messages based on the characteristics of individual recipients to enhance personal relevance and increase attention and message processing. A message might address the recipient by name or reference a personal attribute [60,61]. A tailored message might read as follows:

Your drug use doesn’t define you. You are so much more than that. What other parts of yourself are important to you? What would you want others to know about you? Here’s one you shared with us: “I’m proud that I’m back in school.”

### Conceptual Model

**Figure 2:**
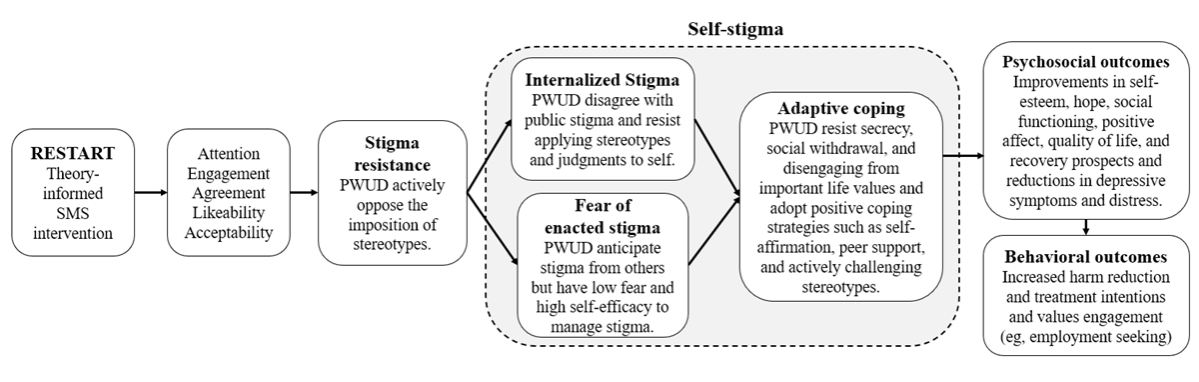
Conceptual model of the intervention. PWUD: people (or person) who use drugs; RESTART: Resisting Stigma and Revaluating Your Thoughts.

The message design and content are informed by health communication and psychotherapeutic theories to increase participants’ attention to, engagement with, and agreement with the messages (Figure 2). We expect the messages will, in turn, improve participants’ stigma resistance by providing information and advice pertaining to its subconstructs, such as challenging stigmatizing thoughts and developing alternative positive identities. Improvements in stigma resistance will give participants the cognitive and behavioral tools to challenge internalized stigma and overcome the fear of enacted stigma [25,44], which will, in turn, improve psychosocial outcomes such as self-esteem and hope [26-30]. Although unmeasured in this pilot study, we expect that reductions in self-stigma from the intervention may attenuate the “why try?” effect and have distal effects on behavioral outcomes such as treatment seeking and harm reduction seeking [18,62-64]. These outcomes could be assessed in future clinical trials if the pilot study demonstrates feasibility, acceptability, and preliminary effectiveness.

### Intervention Development

#### Phase 1: Initial Message Development

Messages were developed using a modified intervention mapping technique [65]. We designated the 4 personal-level domains of stigma resistance as the “change objectives” of the program. Each message was then mapped onto a stigma resistance domain, a “method of change,” (ie, an empirically or theoretically supported approach from the health communication or psychotherapeutic literature), and a commonly held stigma-related belief (to ensure the relevance of the messages’ topics).

An initial bank of 20 messages was developed and refined with an expert committee that included a harm reduction practitioner and researchers with expertise in substance use, stigma, clinical psychology, mobile health (mHealth), and health communication.

#### Phase 2: Intervention Refinement Through Qualitative Elicitation Interviews

We conducted formative qualitative interviews with 22 PWUD to elicit stigmatizing experiences, attitudes, and beliefs; message feedback; and intervention acceptability. Participants were recruited from a syringe service program in Ohio and were eligible if they used illicit drugs in the previous 30 days and owned a mobile phone capable of receiving SMS text messages. Participant ID numbers are included for interview excerpts.

The preliminary 20 messages were tested using concurrent verbal probing on comprehension, liking, relevance, and suggestions for improvement [66]. Each participant reviewed 4 messages. Feedback was iteratively reviewed between interview rounds using the rigorous and accelerated data reduction technique [67], and changes were made for review in subsequent interviews. Overall, each preliminary message was reviewed by 4 to 5 participants. On the basis of participant feedback, messages were simplified to decrease cognitive burden (eg, reducing the reading level or lengthening to clarify content), edited to improve relevance, scaffolded into multiple messages to provide more information, or removed entirely. Several new messages were also created using exemplary in-vivo quotes to raise relevance and credibility, for example:

Carrie gets frustrated that people can’t see past their drug use. “I don’t call you Bill with cancer. I don’t call you Sue with glaucoma. So don’t call me Carrie the addict.” Carrie is not defined by their drug use, and neither are you, [name]. There is so much more to you that makes you... you.

The evolution of select messages is provided in Table 3.

**Table 3:** Examples of message changes from participant feedback.

Original message	Changes	Final message	Exemplifying feedback
“Being an addict isn’t a fair label. If you carry Narcan, some people might call you a guardian angel instead.”	Split the message into 2 to increase information and exposure. Changed label from guardian angel to superhero. Added in-vivo participant quotes.	“MORNING: Steve thinks labels like ‘junkie’ are unfair. He refuses to buy in. It’s like his dad taught him, ‘It’s not what you’re called, it’s what you answer to.’ Can you stand tall and rise above the bullies?” “EVENING: It helps to come up with different labels! Here’s an example: If you carry Narcan, some people might call you a SUPERHERO instead. Does that feel more fair? Be proud of the good you do in the world. Not all heroes wear capes.”	“...maybe I’m your second chance, you know what I mean, I don’t know. I just don’t feel like an angel or guardian angel.” [10.02PA]
“Living with substance use issues is so much harder than most people know. If there’s a silver lining, it’s that only a determined and resilient person could make it this far.”	Changed ‘substance use issues’ to ‘addiction’, which was more salient to participants. Targeted participant (“only a brave and resilient person like YOU”) to increase believability.	“Addiction is so much harder than most people understand. If there’s a silver lining, it’s that only a brave and resilient person like YOU could make it this far.”	Participant: “It’s the ‘only a determined, resilient person can make it this far.’ I don’t believe that. Because a lot of people just get through because it just happens. I don’t know. Divine intervention or just because they rode somebody’s coat tail or something.” Interviewer: “What if the wording changed and said something like you had to be determined and resilient to make it this far. Would that feel better?” Participant: “Yeah. That’d be better.” [09.25PB]
“Our thoughts don’t always match reality. You can tell yourself you’re a pumpkin all day but it won’t make it true. Can we question the negative self-talk, too?”	Because of the high cognitive burden, this message was removed.	Removed	Participant: “That one’s a bit weird.” Interviewer: “Tell me what you think it means.” Participant: “I have no fucking clue. [LAUGHS] That’s gotta be spun. I don’t know.” [10.11PA]
“What are three things you accomplished this week that make you feel proud? How would others feel about you if they knew what you’re capable of?”	Participants enjoyed working through the message and offered no feedback for changes.	Unchanged	Interviewer: “How did it feel to think about it out loud?” Participant: “Actually, it was pretty good. It just refreshes my memory to something that can help me. As far as, which I take that as a tool right now, because I don’t think about it like that, but I just need to remember what I am capable of. That right there will boost my confidence. So that’s a good thing.” Interviewer: “Okay. That’s great. Anything you’d change about it?” Participant: “No, definitely not.” [10.10PB]

Global feedback on the intervention, its content, and delivery was also solicited. Participants tended to prefer personalized messages rather than those that were generic. They also disliked messages that were too abstract or required a lot of cognitive effort and enjoyed messages that suggested future-oriented activities (eg, goal setting). Importantly, several participants noted that messages encouraging social support (eg, sharing something positive with a loved one) would be difficult for PWUD without supportive relationships. These messages were adjusted to offer alternatives, such as writing in a journal or texting the intervention phone number back.

Participants varied in their preferred message frequency. Some participants preferred less frequent messages (“Probably one in a day, maybe” [09.27PB]), whereas others preferred as many as possible (“I don’t care when, how, how many. I would love them.” [09.27PA]). Most participants suggested that about 2 or 3 messages per day would be ideal:

You might send them a message in the morning of, “Good morning, had your coffee this morning?” type of thing. And then some type of, as you go through the day, remember you can start your day over at any time. Don’t let it get to you. [...] And at the end of the day, have you done your gratitude list today before you lay down the night? Are you still thankful for what you got?

09.25PA

#### Phase 3: Finalizing Message Library

##### Overview

The message library was finalized in consultation with the expert committee and includes 56 messages (4 introductory messages, 7-17 messages for each of the 4 stigma resistance domains, and 2 concluding messages). Messages will be delivered in a standard order, emphasizing education and affirmation toward the beginning of the 4-week intervention period and more cognitively demanding content (eg, identifying personal values) toward the end. Sample messages mapped to stigma resistance domains, salient beliefs, and theoretical support are provided in Table 4. Example tailoring strategies are provided in Textbox 1. The message text is simplified to a sixth-grade reading level according to the Simple Measure of Gobbledygook readability formula [68].

**Table 4:** Excerpt from the final message development matrix.

Stigma resistance domain	Salient stigma-related beliefs	Theoretical support	Message
Empowering oneself through learning about substance use and one’s recovery	“I judge myself because I think I can do better.”	Psychoeducation	“TRUE OR FALSE? If you relapse, your recovery is a failure...FALSE! Did you know relapse is common in other chronic conditions like hypertension and asthma? Returning to use is a normal part of recovery from drug use, too. In fact, most people in long-term recovery have had several “relapses.” Even Michael Jordan fell short for six seasons before he won his first championship. Believe in yourself.”
Not believing stigma and catching and challenging stigmatizing thoughts	“When people say bad things about me, I believe them.”	Cognitive restructuring (cognitive behavioral therapy)	“Have you had a bad thought about yourself this week? Let’s think of a reason that thought is NOT true. Here’s an example: ‘My family might think I’m selfish. But that’s not true. To be the best I can be, I have to take care of myself first’.”
Maintaining one’s recovery and proving stigma wrong	“People think I’m never going to change.”	Behavioral activation (cognitive behavioral therapy)	“Haters may say you won’t succeed, but we know you will. You are capable of so much. Let’s do something small today to prove them wrong. Your theme song for today is [song]. Turn it on and get living!”
Developing a meaningful identity and purpose apart from one’s substance use	“I don’t have anything important left in life.” or “I have nothing to look forward to.”	Contact with important values (acceptance and commitment therapy)	“This week, let’s think about your PURPOSE in life. “I was put on planet earth to...” It’s a hard question, but it’s important to think about what keeps us going.”

**Example message tailoring strategies.**

- Demographic tailoring (gender)
  “Did you know (26 million men) or (21 million women) are dealing with a substance use disorder? That’s more than twice the population of Ohio. There is strength in numbers. If someone treats you like you’re different, know that you are not alone in this fight.”
- Personalization (stigmatizing label)
  “OK, here’s the exercise. It sounds silly but give it a try. Ready? Say ‘[word]’ out loud. Over and over. Use a goofy voice. For a full minute. When you’re done, pay attention. Does ‘[word]’ start to lose its meaning? Sometimes it helps to recognize that words are just words. Are you gonna let others put you down with THIS?”
- Personalization (name)
  “Here’s an exercise. What do you want written on your tombstone? ‘[Name] was a good friend, a kind soul, and a brave person’. Now you try. Go ahead and write it down. Don’t lose sight of what makes you worthy.”

##### Orientation

While stigmatizing experiences (ie, stereotyping and discrimination) were salient in the formative qualitative interviews, 12 (55%) of 22 participants were unfamiliar with the meaning of “stigma.” In response, we developed a brief primer on stigma to share during the enrollment phase of the pilot trial.

#### Preliminary Acceptability

Acceptability of the intervention was high in the formative interviews:

I think it would be great. [...] You could be at the worst moment and something like that come up and it could turn your whole day around, you know, or turn your decision around or, you know, whatever you’re going through.

10.02PB

I think it would be great for real. It shows that people care that don’t have to. And that matters. [...] I mean the program itself, to do something like that is awesome. [...] It’s the people that care that don’t have to.

09.25PB

Even if this program don’t change the world to some of us addicts, it may birth the next program that does.

10.02PA

Even participants who admitted coming into the interviews skeptical of the research or unhappy with previous counseling experiences conceded that the program might have value:

So honestly, I think it’s good. I’ll be honest. At first I was like, this is gonna be stupid. All these stupid rehabs around here and shit. I was. I’m for real I was. I feel like, cliche. Like, I mean, that’s what I thought before I came and stuff. And then I thought even like, OK, it’s going to be cliche. But, you know, not snobby, but like, another... but no, that’s very positive. It really is. I have not heard anyone say stuff or do anything like this in [town name].

10.16PA

I mean, some of it might be corny, but then so what? Some of it’s corny, but some of it ain’t. You know what I mean? So I don’t think that I would look at it like that. [...] I don’t feel like it’s coming from somebody that’s a stranger that’s just there for a paycheck. [...] We don’t get to hear positive things a lot. [...] We don’t say them to ourselves, nor do we get to hear them from others. So I think it’s a good idea.

10.12PA

Several participants expressed enthusiasm to participate even without compensation, noting the anticipated value of the program to themselves or other PWUD. Though all participants were prompted for concerns, only 1 was offered:

I don’t think it’s a bad idea at all[...] Maybe like the only concern I would have is for like girls who are in relationships with guys[...] Because there’s always that, well, who’s calling you? Who’s texting you?

10.02PB

The overwhelmingly positive feedback made us confident to proceed with a pilot trial.

### Study Design for the Pilot Trial

#### Overview

We will assess the feasibility, acceptability, and preliminary effectiveness of RESTART in a pilot trial using a 1-group prepost within-subjects design [69]. All participants will be assigned to the intervention. Preliminary effectiveness measures will be captured at baseline and follow-up via self-report. Demographics will be captured at baseline only. Feasibility and acceptability measures will be captured at follow-up only.

#### Setting and Participants

The setting for this pilot trial is a rural county in southern Appalachian Ohio designated as economically distressed by the Appalachian Regional Commission [70]. Southern Ohio has been an epicenter of the overdose epidemic for the past 2 decades, with morbidity and mortality rates consistently outpacing the rest of the state and nation [71,72]. High rates of substance use in this region are explained, in part, by high rates of disability from blue-collar jobs, a proliferation of “pill mills” (clinics that prescribe opioids inappropriately for financial gain) early in the epidemic, low evidence-based treatment capacity, and social and economic disadvantage in the region [73-78].

Participants will include 30 PWUD residing in the study county. Previous guidance has indicated that our sample size is an acceptable number for feasibility studies [79-81]. This sample size will allow us to estimate the retention rate to within a 95% CI of +12% or –12% (calculated assuming a retention rate of 85%). The sample size will further allow us to detect moderate effect sizes in the continuous outcomes (Cohen *d*=0.53) with 80% power (α=.05, 2-tailed) using paired *t* tests [82].

#### Eligibility and Recruitment

Prospective participants will be recruited from a local harm reduction program. The program has been active since 2011 and has cultivated a high level of trust with its clients, serving approximately 2000 individuals annually and supporting multiple research studies since its inception. Participants will also be identified through referral by enrolled participants and from contact lists of research participants consenting to recontact from previous studies. To be eligible, participants must be aged at least 18 years; understand English; have reliable daily access to a smartphone with a data plan capable of sending and receiving SMS text messages; reside in the study county with no immediate plans to move; and report past-30 days’ use of illicit opioids (eg, heroin and fentanyl), prescription opioids not as prescribed (eg, oxycodone, buprenorphine), methamphetamines, or cocaine. To prevent ineligible individuals from attempting to participate, dummy screening questions will be included to obscure the eligibility criteria, as familiarity with research is high in the community. Participants will be purposively recruited to approximate the gender and racial and ethnic diversity of the study county.

#### Enrollment

Participants will provide consent and be enrolled in person, and then, they will complete a baseline survey via Computer-Assisted Self-Interview using the Qualtrics survey management system (version March 2024; Qualtrics LLC) [83]. The survey is expected to take 45 to 60 minutes to complete. Participants will then be oriented to the intervention by study staff on study procedures, phone security, handling technical issues, and setting up their phone to receive messages. Study staff will then provide a brief primer on stigma, including its historical background, definition, components (ie, stereotypes, prejudice, and discrimination), forms (eg, public stigma and self-stigma), and consequences (eg, social avoidance). Finally, participants will provide their personalization preferences, including message timing, preferred name, and other details that will be incorporated into tailored message templates.

#### Message Delivery

Participants will receive 2 messages per day (morning and evening) for 28 consecutive days, beginning on the day after enrollment. Messages will be delivered automatically via Twilio (version April 4, 2010; Twilio Inc), a communication platform that uses end-to-end encryption and follows industry-standard security measures. Automation is programmed in Python (version 3.11; Python Software Foundation), a programming language that is compatible with Twilio’s application programming interface. Python scripts, message libraries, phone number databases, and other files needed for automation will be stored in AWS Lambda (Amazon Inc), Amazon’s serverless computer service for running code. The program will be triggered to run every minute with Amazon EventBridge Scheduler (Amazon Inc).

#### Follow-Up

Participants will be contacted a few days before their scheduled end date to come in for their follow-up Computer-Assisted Self-Interview survey. The follow-up survey is expected to take approximately 60 minutes to complete. A subsample of participants (12/30, 40%) will also be selected to complete a brief interview covering experiences with and opinions of the program. Interview participants will be randomly selected from those presenting for their follow-up visit.

#### Measures

##### Implementation Feasibility

**Figure 3:**
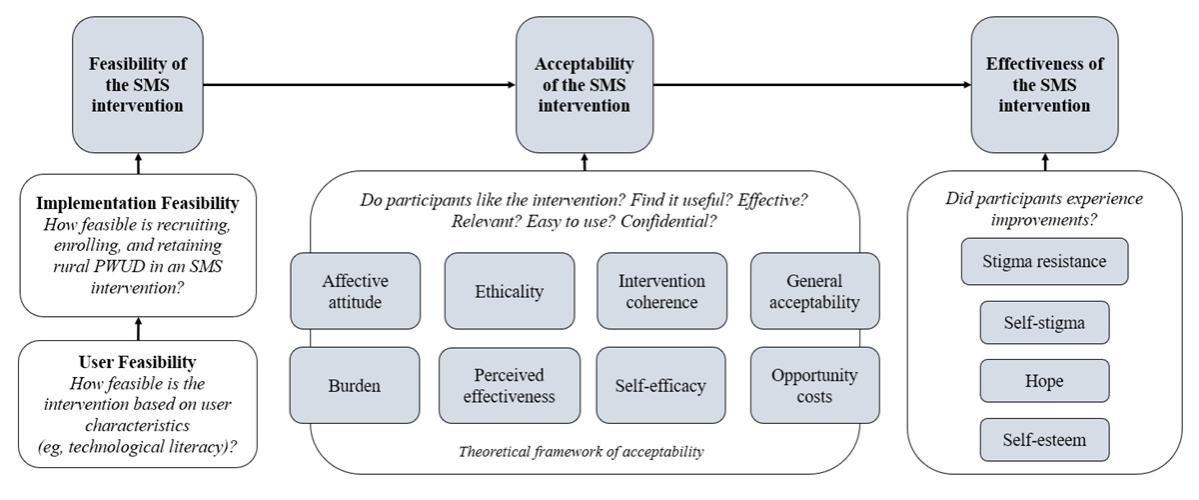
Conceptual model of the feasibility, acceptability, and effectiveness measures. PWUD: people (or person) who use drugs.

The feasibility of implementing the intervention will be assessed through recruitment, enrollment, retention, and message delivery statistics (Figure 3). We will measure the percentage of contacted individuals who proceed to screening, the percentage of screened individuals who are eligible, the percentage of eligible participants who enroll in the study, the time to reach sample saturation, the dose delivered (ie, percentage of messages successfully sent), and the dose received (ie, percentage of messages successfully delivered to the participant’s phone per the carrier network). Read receipts are unavailable for standard SMS text messages delivered through Twilio.

##### User Feasibility

Intervention feasibility from users’ perspectives will be measured at follow-up using three 5-point Likert-type items and 2 binary (yes or no) items adapted from the study by Lian et al [84]:

Before the start of this program, how often did you send or receive text messages?

Before the start of this program, how comfortable were you with sending or receiving text messages?

Between the start of the program and now, did you experience any challenges or changes with your phone (e.g., lost, broken) that prevented you from receiving or reading text messages?

Between the start of the program and now, did you experience any challenges or changes with your phone number or phone plan (e.g., changed number, ran out of minutes) that prevented you from receiving or reading text messages?

On average, when would you read the text message you received?

Follow-up interviews will provide additional context by probing how participants engaged with the program, whether technical issues arose, and suggestions to make the program easier to use.

##### Acceptability

Our literature review availed no standardized measures to assess acceptability in the context of a passive SMS text message intervention. We constructed a novel 18-item Likert-type questionnaire to assess acceptability based on the Theoretical Framework of Acceptability [85] with items adapted from Sekhon et al [86], Lian et al [84], and Knutson et al [87]. Items address the following Theoretical Framework of Acceptability domains: affective attitude (eg, “Overall, how much did you like or dislike the text message program?”), burden (eg, “How much effort did it take to engage with the text message program?”), ethicality (eg, “I had concerns about the privacy of my information sent over text message”), intervention coherence (eg, “It is clear to me how the text message program can help me to deal with stigma”), opportunity costs (eg, “Engaging with the text message program interfered with my other priorities”), perceived effectiveness (eg, “Overall, how helpful did you find the program?”), self-efficacy (eg, “How confident did you feel about using the information in the text messages?”), and general acceptability (“How satisfied were you with participating in this study?”). Follow-up interviews will provide additional context by probing on messages and intervention features participants liked or disliked, program concerns, and suggestions for improvement.

##### Preliminary Effectiveness

Primary effectiveness outcome measures are self-stigma and stigma resistance. Self-stigma will be assessed using the 40-item Substance Abuse Self-Stigma Scale (scale range 40-200; α=.86), which includes 4 subscales: self-devaluation, fear of enacted stigma, stigma avoidance, and values disengagement [25]. Stigma resistance will be assessed with the 20-item Stigma Resistance Scale (scale range 20-100; α=.93; subscales: self-other differentiation, personal identity, personal cognitions, peer stigma resistance, and public stigma resistance) [88]. Secondary effectiveness measures are hope and self-esteem, assessed using the 12-item Adult Dispositional Hope Scale (scale range 8-64; α=.77-.84), which measures agency and pathways to meet one’s goals, and the 10-item Rosenberg Self-Esteem Scale (scale range 10-40; α=.77), which measures general self-esteem [89,90]. All scales are summed and scored such that greater scores reflect more favorable outcomes, except for the Substance Abuse Self-Stigma Scale, for which lower scores reflect lower self-stigma. In addition, once per week, participants will be texted a URL to a Qualtrics survey to rate the perceived message effectiveness of the previous week’s messages using a single Likert-type item (“How much did each message help you understand how you can cope with stigma?”). Follow-up interviews will provide additional context on changes in knowledge and attitudes during the program.

#### Analysis

Basic descriptive statistics will be calculated for feasibility, acceptability, and outcome measures. We will present frequency tables for the categorical variables and means, SD, and percentiles (25th, 50th, and 75th) for the continuous variables. For outcome measures, descriptive statistics will be calculated for baseline scores, follow-up scores, and changes in scores.

As this is a feasibility study underpowered for statistical inference, hypothesis testing on the effectiveness outcomes is strictly exploratory. The analysis assumes a null hypothesis of no participant-level change in the outcome measures from baseline to follow-up. Inferential analyses will be conducted on participant-level change in outcome measures from baseline to follow-up using paired *t* tests (or Wilcoxon signed rank tests if response distributions are nonnormal) [69]. We will also examine differences in mean change scores across levels of demographic variables using ANOVA [69]. We will follow an intention-to-treat approach for all outcome measures. Significance levels for analyses will be set at α=.05. We will use R software (version 4.3.0; The R Foundation) for data cleaning, management, and analysis.

## Results

Recruitment and enrollment began in March 2024. Follow-up visits concluded in April 2024. Data analyses were completed in July 2024. Findings from this study, regardless of outcomes, will be documented in manuscripts and submitted to relevant peer-reviewed journals. Results will also be reported on ClinicalTrials.gov (NCT06281548).

## Discussion

### Overview

In this paper, we described the process of developing, implementing, and evaluating a novel automated SMS text message intervention for PWUD. This intervention leverages the theory of stigma resistance with support from the psychotherapeutic and health communication literature to reduce substance use stigma. Messages were designed by researchers with expertise in stigma, substance use, clinical psychology, health communications, and mHealth. Importantly, the intervention received critical input from PWUD on message content, format, tailoring, timing, and frequency. Our study design will allow us to explore multiple quantitative and qualitative facets of feasibility, acceptability, and effectiveness, ultimately informing whether to proceed with a larger clinical trial.

### Principal Findings

To the best of our knowledge, this is the first study to address substance use stigma via a self-help SMS text messaging modality. SMS text messaging interventions with promising effectiveness have been implemented to support individuals with mental health conditions, including depression, schizophrenia, eating disorders, and substance use [91]. SMS text messaging is cost-effective and scalable, with fewer technical challenges and equity concerns than mobile apps [92]. Cope Notes is one exemplar that sends automated supportive text messages with evidence for reducing anxiety and depressive symptoms [93]. The creators claim to have 33,766 subscribers across 97 countries as of March 2024 [94]. RESTART provides a test case for extending the potential of SMS text messaging interventions to PWUD, a historically hard-to-reach and hardly reached population [95].

Despite widespread acknowledgment that stigma is a key barrier to harm reduction and treatment use, few studies have intervened on this construct among PWUD in active use [32]. The results of our study will address the current research gap in stigma reduction interventions for PWUD. Our study will further inform whether SMS text messaging is a feasible and acceptable modality for promoting preventive health behaviors in this population.

### Limitations and Anticipated Challenges

Our study has several limitations and potential challenges. The modest sample size and quasi-experimental design limit our ability to detect effects and make causal inferences. However, the primary aim of the study is to determine the feasibility and acceptability of both the intervention and study design; effectiveness may be further explored in future trials. The intervention was informed by and will be evaluated in PWUD residing in rural southern Ohio, limiting the generalizability of our findings. Retention is a challenge in studies with PWUD, although we have included multiple staggered incentives to minimize this risk [96,97]. Our harm reduction partners have also informed us that many clients frequently change phone numbers or data plans. Participants will be instructed to contact the study team if such technical issues arise, and alternate contact information will be obtained in cases where participants are lost to follow-up. Finally, although not shared by our qualitative participants, privacy and confidentiality may be concerns, particularly given the sensitive nature of the messages. Our communication platform, Twilio, has been used in multiple mHealth studies and uses industry-standard encryption algorithms [98-100]. Participants will also be instructed at enrollment to password protect their phones and provided advice on avoiding inadvertent disclosure.

### Conclusions

Almost all federal funding allocated in the United States to date addresses public stigma through mass media campaigns and education programs, although there are questions about the extent to which these approaches move the needle [24,101-104]. Findings from this proposed study will add to the evidence base for self-stigma reduction as a necessary complement to public-facing initiatives in the stigma reduction toolkit [24,32]. Preliminary evidence from our formative qualitative study suggests that RESTART may be an acceptable approach to providing information and advice on self-stigma to PWUD. Our pilot intervention will provide further evidence of acceptability, feasibility, and preliminary effectiveness in anticipation of a scale-up clinical trial. This protocol may be of interest to researchers considering an SMS text messaging modality to address psychosocial needs in hard-to-reach populations such as PWUD.

## Appendix

Multimedia Appendix 1 Peer review report from the National Institute on Drug Abuse.

## Abbreviations

CBT: cognitive behavioral therapy
mHealth: mobile health
PWUD: people (or person) who use drugs
RESTART: Resisting Stigma and Revaluating Your Thoughts
UNC: University of North Carolina-Chapel Hill

## Data Availability

The data sets generated and analyzed during this study are available from the corresponding author on reasonable request.
