# Supplementary material for: An Automated Text Messaging Intervention to Reduce Substance Use Self-Stigma (Project RESTART): Protocol for a Feasibility and Acceptability Pilot Study
Source: JMIR Res Protoc. 2024 Aug 9;13:e59224. doi: 10.2196/59224 (PMC11344186; doi:10.2196/59224)
Supplement: Multimedia Appendix 1 [file resprot_v13i1e59224_app1.pdf]

SUMMARY STATEMENT
(Privileged Communication)

PROGRAM CONTACT:
Richard Jenkins
301-443-6504
jenkinsri@mail.nih.gov

Release Date: **03/31/2023**
Revised Date:

Application Number: **1 F31 DA058452-01A1**
Formerly: **1F31DA058452-01**

SIBLEY,ADAMS
University of North Carolina at Chapel Hill
302 Rosenau Hall; CB#7440
Chapel Hill, NC 275991350

Review Group:	**ZRG1 F16-L (20)**<br>**Center for Scientific Review Special Emphasis Panel**<br>**Fellowships: Clinical Care and Health Interventions**
Meeting Date:	**03/06/2023**
Council:	**MAY 2023** ***PCC:*** **CV/RJP**
Requested Start:	**07/01/2023**

Project Title:	**Determining the Feasibility and Acceptability of a Novel Stigma Resistance Text Message Intervention for People who Use Drugs**<br>***Requested*: 2 Years**
Sponsor:	**Go, Vivian**
Department:	**Health Behavior**
Organization:	**UNIV OF NORTH CAROLINA CHAPEL HILL**
City, State:	**CHAPEL HILL NORTH CAROLINA**
SRG Action:	**Impact Score:10 Percentile:2 +**
Next Steps:	**Visit https://grants.nih.gov/grants/next_steps.htm**
**Human Subjects:**	**30-Human subjects involved - Certified, no SRG concerns**
**Animal Subjects:**	**10-No live vertebrate animals involved for competing appl.**
**Gender:**	**1A-Both genders, scientifically acceptable**
**Minority:**	**1A-Minorities and non-minorities, scientifically acceptable**
**Age:**	**3A-No children included, scientifically acceptable**

1F31DA058452-01A1 Sibley, Adams

RESUME AND SUMMARY OF DISCUSSION: This resubmitted predoctoral fellowship application requests support for training in behavioral health to conduct research that facilitate the development and testing of an mHealth intervention to reduce stigma among individuals who use drugs. This was a highly responsive resubmission. During the discussion, the reviewers noted many strengths of the applicant including strong research experiences using community-engaged approaches, prior collaboration and engagement with the community, strong academic performances, and outstanding letters of support. The mentoring team was noted to be strong with necessary experience and complementary expertise to guide the applicant to successful completion of the research and training activities proposed. The training environment was noted to be supportive and suitable. The training plan was noted to be strong and complementary to the candidate's training needs and included activities to broaden the candidate's skills and expertise. The research plan was noted as significant and innovative with the development of an mHealth intervention. Some minor and addressable weaknesses were raised around that limited justification for the proposed sample size for study aim four and the limited attention to racial ethnic diversity in the proposed sample. There were differences of opinion among reviewers regarding whether a DSMB would be required for this project given the work with vulnerable populations. Weaknesses noted did not detract from the overall impact of the application. Following the discussion, the panel agreed that the strengths of a strong applicant, a competent mentoring team, and robust research and training activities will contribute to the candidate having a high potential to be successful as an independent investigator.

DESCRIPTION (provided by applicant): Nearly 850,000 Americans have died from overdose in the past two decades. Stigma remains an under- addressed central barrier to ending the drug epidemic. Substance use stigma reduces treatment and harm reduction utilization among people who use drugs (PWUD) and is associated with increased drug use, depression, social isolation, and numerous other psychosocial indicators of healthy functioning. Though interventions focused on education and social contact have proven successful in reducing stigmatizing attitudes in the public, much less is known about how to intervene on self-stigma, the negative feelings PWUD have about their drug use. Developing strategies for PWUD to cope with and resist stigma may promote quality of life and reduce overdose in this population. Stigma resistance is a promising approach that promotes resilience through self-empowerment and positive identity formation. In line with NIDA's priorities of reducing stigma and developing recovery support strategies, the goal of this application is to develop and evaluate a stigma resistance text message intervention for PWUD. Nested within the NIDA-funded Ohio Opioid Project (UG3/UH3DA044822), the specific aims of the proposal are to: 1) Identify PWUD self-stigma subgroups and describe associated demographic, health, and drug use risk factors among rural PWUD in an eight-state multi- site cohort using latent class analysis; 2) identify stigma-related attitudes and beliefs and text message content and delivery preferences through iterative elicitation interviews with 20 rural Ohio PWUD; 3) develop a four- week automated daily text message stigma resistance intervention; and 4) evaluate the feasibility, acceptability, and preliminary effectiveness of the intervention in a sample of 30 rural Ohio PWUD in active use. An iterative convergent mixed-methods design informed by health communication and behavioral health theories will guide the development of interactive messages that are hypothesized to increase stigma resistance and reduce self-stigma. This proposal will lay the groundwork for a self-stigma intervention and provide further evidence for text messaging as a cost-effective, impactful modality for engaging this hard-to- reach population. Alongside the planned research, the applicant proposes training that will enhance his theoretical understanding of stigma, advance his quantitative analysis skills, strengthen his competence in health communication strategies, and build his communication record through scholarly presentations and publications. Under the expert mentorship of co-sponsors Drs. Vivian Go and William Miller and other collaborators, this

research and training plan will contribute to the applicant's career trajectory as an independent mixed-methods substance use and stigma researcher.

PUBLIC HEALTH RELEVANCE: Self-stigma is a key barrier to help-seeking during the opioid epidemic, especially in rural areas, yet there are remarkably few evidence-based interventions to address this phenomenon. The present research uses a behavioral theory-informed, mixed-methods approach to develop and evaluate a novel text message intervention to promote resilience to stigma and reduce feelings of self-stigma among rural people who use drugs. Findings will add to scientific knowledge about feasible and acceptable stigma reduction interventions and provide further evidence of whether text messaging is a productive, cost-effective approach for engaging this hard-to-reach population.

CRITIQUE 1

Fellowship Applicant: 1
Sponsors, Collaborators, and Consultants: 1
Research Training Plan: 2
Training Potential: 1
Institutional Environment & Commitment to Training: 1

Overall Impact:/Merit: The applicant is a 3rd year PhD student in Health Behaviors at UNC-Chapel Hill. The applicant had 6 years of experience working in an academic community-engaged research center that collaborated with regional government on prisoner reentry and youth violence prevention, and now in graduate school has several years of high-level involvement in a large community-engaged research project that focuses on opioid overdose prevention in Appalachia. Letters of support highlight extraordinary motivation and advanced analytic and leadership skills. The applicant has a good track record of publications. The strong mentorship team has expertise in stigma research, health communication, mHealth interventions, and the primary sponsor has good mentorship experience (4 K awards, 2 T32 trainees). The research project focuses on developing and pilot testing an mHealth text message intervention that is tailored to the needs of rural persons who use drugs (PWUD). Significance is high because few studies have established how to address self-stigma and research on stigma resistance will advance the field. The sponsorship team has the expertise to guide the applicant and the project is aligned with the training plan. Training will include formal coursework (e.g., usability testing for mHealth interventions), self-directed learning (e.g., programming with Python language), and meetings with sponsors and co-sponsors. This is a resubmission, which was responsive to the prior mostly minor critiques by paring down the number of planned courses and teaching duties. Overall, this is an outstanding application from an accomplished student who is already immersed in the field of opioid overdose prevention and is able to articulate a clear and compelling plan for achieving research independence. The potential for impact in the field is very high.

1. Fellowship Applicant:

Strengths

- Letters of support and sponsor statement categorize the applicant as one of the most talented students they have ever worked with.
- Strong foundation in qualitative research and recognizes the need to bolster quantitative and mixed methods skills.

- Demonstrates excellent understanding of community needs in regards to modifiable barriers to SUD treatment or harm reduction interventions. Likely stems from years working in this field and experience with community-engaged research.
- Already has grant writing experience – wrote a COVID supplement for the sponsors' UG3/UH3 grant.
- Six publications during graduate training

Weaknesses

- None noted.

2. Sponsors, Collaborators, and Consultants:

Strengths

- The Sponsor, Dr. Vivian Go, has expertise in SUD-related stigma and has mentored 13 doctoral students, 8 post-doctoral fellows, and 6 junior faculty members. She has supervised 4 students on F31 awards.
- Dr. William Miller is an infectious diseases physician and epidemiologist with extensive experience in implementation research and research mentorship.
- Dr. Nisha Gottfredson will provide expertise in quantitative methods.
- Dr. Kate Muessig will provide expertise in mHealth intervention design and evaluation.
- Dr. Seth Noar will provide expertise in health communications and the text messaging intervention. The applicant has published with Dr. Noar previously.

Weaknesses

- None.

3. Research Training Plan:

Strengths

- Strong rationale for focusing on self-stigma, good theoretical underpinning for the tailored intervention.
- Conceptualizing substance use stigma and developing interventions to build stigma resistance and mitigate self-stigma (or internalized stigma) will provide a meaningful project and opportunities for future funding.
- An mHealth/text message intervention will be low burden for participants, and may not reach 100% of PWUD, but could be a good addition to the tools used to engage PWUD who have experienced discrimination in healthcare settings.
- Sponsor's Ohio Opioid Project, the parent grant, is focused on community-level interventions to reduce opioid overdose deaths, and will provide an outstanding platform for the planned intervention development component of the study that the applicant has proposed.
- Using Latent Class Analysis to identify unobserved groups based on stigma measures in the parent study will aid in tailoring stigma messages. There will also be in-depth qualitative interviews with PWUD to adapt specific messages.
- Pilot testing of the mHealth/text message intervention will include concrete measures of feasibility, acceptability and usability.

- Applicant will gain experience with stigma resistance and self-stigma measures.

Weaknesses

- There is a lot of work proposed for a 2-year fellowship, considering the amount of ongoing coursework that is also proposed. There will only be 4 months for intervention pilot test recruitment and enrollment.

4. Training Potential:

Strengths

- Strong theoretical rationale for the tailored intervention, applicant conveys mastery of health behavior concepts that will be incorporated into this work.
- There will be coursework in mHealth interventions for Behavior Change and usability testing of digital products. A prior reviewer questioned the need for coursework post-qualifying exam, but I think this will be another outstanding opportunity for the applicant to deepen knowledge and complement applied learning experiences. The mentorship team also has technical expertise to guide the applicant through development of the mHealth intervention.
- Statistical modeling coursework will be applied in the research project, where the applicant will use Latent Class Analysis to categorize stigma sub-groups.
- Developing the text messaging intervention should lead to future opportunities for scholarship.

Weaknesses

- None.

5. Institutional Environment & Commitment to Training:

Strengths

- UNC offers desired coursework in mHealth intervention and Multimedia design.
- Communicating for Health Impact Lab directed by Dr. Noar is a unique resource and ideal for the planned activities.
- Collaboration within the Rural Opioid Initiative will provide future opportunities.

Weaknesses

- None.

Protections for Human Subjects:

Acceptable Risks and Adequate Protections

- Potential for breach of confidentiality.

Data and Safety Monitoring Plan (Applicable for Clinical Trials Only):

Acceptable

  - Will be included within parent study's DSMP

Inclusion Plans:

- Sex/Gender: Distribution justified scientifically

- Race/Ethnicity: Distribution justified scientifically
- For NIH-Defined Phase III trials, Plans for valid design and analysis: Not applicable
- Inclusion/Exclusion Based on Age: Distribution justified scientifically
- Will attempt outreach to recruit Latinx participants.

Vertebrate Animals:

Not Applicable (No Vertebrate Animals)

Biohazards:

Not Applicable (No Biohazards)

Resubmission:

- Critiques were mostly minor relating clarifying gaps in skills, detailing content of meetings with sponsors, and reducing teaching and coursework. The revised application was responsive.

Renewal:

Not applicable

Training in the Responsible Conduct of Research:

Acceptable

Comments on Format (Required):

- Grad school courses and web-based CITI training.

Comments on Subject Matter (Required):

- Additional coursework on research ethics.

Comments on Faculty Participation (Required):

- There will be discussions with advisors.

Comments on Duration (Required):

- Weekly meetings with advisors, 14-hour research ethics course.

Comments on Frequency (Required):

- Will need CITI refresher courses in 2023 and 2024.

Applications from Foreign Organizations:

Not Applicable

Select Agents:

Not Applicable (No Select Agents)

Resource Sharing Plans:

Not Applicable (No Relevant Resources)

Authentication of Key Biological and/or Chemical Resources:
Not Applicable (No Relevant Resources)

Budget and Period of Support:
Recommend as Requested

CRITIQUE 2

Fellowship Applicant: 1
Sponsors, Collaborators, and Consultants: 1
Research Training Plan: 3
Training Potential: 1
Institutional Environment & Commitment to Training: 1

Overall Impact:/Merit: The applicant seeks to develop and evaluate a stigma resistance text message intervention for people who use drugs (opioids). The candidate's training goals include developing a strong understanding of the theoretical underpinnings of stigma in context of substance use, develop expertise in advanced-level statistics, such as LCA, mHealth development and health communication interventions, and build publication record. The applicant has assembled a well-qualified mentoring team, with significant experience mentoring and training doctoral students and postdoctoral fellows. The applicant is also strong, with significant research and leadership experience, albeit with a modest publication record. I was enthusiastic about the research proposal and the training potential of this applicant.

1. Fellowship Applicant:
Strengths
- Has a stated overarching research goal of improving the health and well-being of people who use drugs and has worked towards that goal through choice of employment as an academic community-engaged research coordinator.
- Excellent academic background, indicating a commitment to education and achieving career goals.
- Modest publication record; four publications (2 first authors); one in Lancet Regional Health.
- Strong letters of recommendation; one referee lauded the applicant's detailed approach to the data analysis process.

Weaknesses
- None.

2. Sponsors, Collaborators, and Consultants:
Strengths

- Dr. Go is a social epidemiologist and implementation scientist with expertise in substance use treatment; Dr. Go has considerable experience mentoring students in HIV, implementation science, and SUD research; 25 doctoral students and 8 postdocs.
- Dr. Go is funded and will provide the resources needed for the applicant to complete the study; expertise on stigma reduction will be useful for mentoring/training of the applicant.
- Dr Miller has advised 37 doctoral students and serve on 48 dissertation committees, thus adding to the strength of the mentoring team; has established working relationship with the applicant's primary sponsor; has focused on substance use in Appalachia.
- Dr. Gottfredson will provide expertise in EMA and higher-level statistical analysis training, Dr. Noar, an expert in health communication, will provide training in communication theory and message development, Dr Muessig will provide expertise in the design of mHealth.

Weaknesses

- None noted.

3. Research Training Plan:

Strengths

- Stigma, while recognized as an important barrier to ending the opioid epidemic, is understudied. Understanding what self-stigma looks like among people who use drugs, including attitudes and beliefs, and which ones can be changed can potentially be one avenue to addressing the opioid crisis. Thus, the proposed study is of significant public health interest and, if successfully conducted, can be of high impact.
- Use of SMS as part of an mHealth treatment is practical and can be of broad reach, thus also increasing the potential impact of the study.
- The proposed study is based on sound theoretical frameworks and conceptual models.
- The proposed study is nested in an ongoing study, the Ohio Opioid Study (which is one site in a larger multisite study)
- The study team has established relationships and trust with SHRPS, a syringe service program where participants will be recruited from.
- Data analysis plan are thorough and well justified.

Weaknesses

- It appears that the applicant will be doing a lot of the research activities. This gives me slight concern about feasibility.
- The proposed sample is disproportionately white thus limiting the impact of the study.

4. Training Potential:

Strengths

- Sponsors and other members of the mentoring team are engaged in the applicant's training and the proposed study.
- Applicant has provided clear training goals and delineated how these goals will be met.
- Candidate proposes to submit three dissertation manuscripts for publication.
- Knowledge gaps are clearly delineated, and training needed to gain more knowledge described.

Weaknesses

- No concerns.

5. Institutional Environment & Commitment to Training:

Strengths

- Gillings School of Global Public Health is highly rated, with strong emphasis on community engagement, which is integral to research in the applicant's area of interest.
- The Odum Institute for Research in Social Science provides courses and workshops, which can be utilized by the applicant.
- UNC Injury Prevention Research Center supports and conducts research in opioid addiction and overdose and provide mentorship, expertise, and resources.

Weaknesses

- None noted.

Protections for Human Subjects:

Acceptable Risks and Adequate Protections

Data and Safety Monitoring Plan (Applicable for Clinical Trials Only):

Unacceptable

- o No information was provided about DSMB. Given that an intervention will be offered, a DSMB should be given.

Inclusion Plans:

- Sex/Gender: Distribution justified scientifically
- Race/Ethnicity: Distribution not justified scientifically
- For NIH-Defined Phase III trials, Plans for valid design and analysis: Not applicable
- Inclusion/Exclusion Based on Age: Distribution justified scientifically

Vertebrate Animals:

Not Applicable (No Vertebrate Animals)

Biohazards:

Not Applicable (No Biohazards)

Resubmission:

- Generally responsive to previous.

Renewal:

Not applicable

Training in the Responsible Conduct of Research:
Acceptable
Comments on Format (Required):
Comments on Subject Matter (Required):
Comments on Faculty Participation (Required):
Comments on Duration (Required):
Comments on Frequency (Required):

Applications from Foreign Organizations:
Not Applicable

Select Agents:
Not Applicable (No Select Agents)

Resource Sharing Plans:
Not Applicable (No Relevant Resources)

Authentication of Key Biological and/or Chemical Resources:
Not Applicable (No Relevant Resources)

Budget and Period of Support:
Recommend as Requested

CRITIQUE 3

Fellowship Applicant: 1
Sponsors, Collaborators, and Consultants: 1
Research Training Plan: 2
Training Potential: 1
Institutional Environment & Commitment to Training: 1

Overall Impact:/Merit: In this revised application, the applicant has strengthened a research training plan that was generally viewed favorably by reviewers. The relatively small number of concerns have been addressed effectively through clearer articulation of training goals and activities and clearer linkage between the training activities and the proposed research. The result is a strong application with substantial promise as described in the uniformly positive comments from sponsors and in the letters of recommendation. The Sponsor and Co-Sponsor have strong publication, funding, and mentorship records and have collaborated for more than a decade on research and training in areas close to the focus of the application. The research plan is sound and will lay the groundwork for high-impact work by the applicant after completing graduate studies. The two-year training plan is thorough and cohesive, and will be implemented in an outstanding training environment. Overall, it is likely that the activities

outlined in the application will position the applicant for research independence and productivity after graduate training is completed.

1. Fellowship Applicant:

Strengths

- The applicant worked six years as academic community-engaged research coordinator, developing skills that will contribute to the proposed research.
- The applicant has been productive, contributing to six peer-reviewed publications (four from the parent project); four more are in progress (applicant is first-author on two focused on stigma).
- The applicant's academic record is strong. including multiple PhD courses that provide a foundation for the proposed training activities.
- Letters emphasize the applicant's research skills and commitment to the proposed training and research.

Weaknesses

- None noted.

2. Sponsors, Collaborators, and Consultants:

Strengths

- The Sponsor and Co-Sponsor have worked together on funded research for more than a decade; their collaborative work supports trainees at both institutions.
- Both sponsors have very strong track records of garnering grant support and placing papers in high impact journals.
- The Co-Sponsor, though not at the applicant's institution, interacts with the applicant often as part of their collaborative work on the parent grant.
- The mentorship team is well chosen, offering the applicant access to high-level expertise in health messaging, intervention design, and advanced statistical modeling.
- The sponsors have ample funds to support the research and training activities.

Weaknesses

- None noted.

3. Research Training Plan:

Strengths

- The applicant is well positioned to reach a high-risk population, rural opioid and injection drug users, ensuring that information that shapes the intervention will be relevant and tailored to the intervention target group.
- The proposed research takes advantage of the sponsors' and the applicant's key roles in the parent study, which has produced evidence of pervasive self-stigma.
- Technical aspects of the mHealth intervention development are well described and will benefit from expertise provided by members of the mentor team.
- The goal of reducing stigma as a step toward reducing the negative consequences of use or harm reduction strategies is innovative and important.

Weaknesses

- The preliminary intervention will enroll a small number of participants, precluding meaningful comparisons of subgroups such as those identified by the latent class analyses.

4. Training Potential:

Strengths

- The long-term goal of becoming an independent mixed methods substance use researcher with an emphasis on stigma against users is clearly articulated by the applicant and sponsors, and the beginning of its pursuit is clearly outlined in the research and training plans.
- The sponsors and members of the mentor team are well equipped to oversee and participate in achieving the training goals.

Weaknesses

- None noted.

5. Institutional Environment & Commitment to Training:

Strengths

- The environments at UNC and Ohio State are very strong and will provide all of the resources needed for achieving the training and research goals.

Weaknesses

- None noted.

Protections for Human Subjects:

Acceptable Risks and Adequate Protections

- For focus groups, recordings deleted after transcription; identifying info removed from transcripts.
- Minor risk of distress, but participants can choose not to answer any question; will be provided list of local counseling services.
- All staff trained in confidentiality protection; any confidentiality issues discussed at weekly staff meetings.
- Research staff will ensure participants' phones are password protected so no one can get to their text messages.

Data and Safety Monitoring Plan (Applicable for Clinical Trials Only):

Not Applicable (No Clinical Trials)

Inclusion Plans:

- Sex/Gender: Distribution justified scientifically
- Race/Ethnicity: Distribution justified scientifically
- For NIH-Defined Phase III trials, Plans for valid design and analysis: Not applicable
- Inclusion/Exclusion Based on Age: Distribution justified scientifically

- Children were not included in the parent study and are therefore excluded; no exclusion for adults.
- No restriction for gender; approximately equal proportions women and men.
- No exclusion on race/ethnicity; additional efforts to recruit Hispanic/Latinx participants to increase sample representativeness.

Vertebrate Animals:

Not Applicable (No Vertebrate Animals)

Biohazards:

Not Applicable (No Biohazards)

Resubmission:

- Added missing descriptions of how training plan addresses current gaps and is connected to aims.
- Information is provided about interactions with sponsors and consultants with reference to training goals.
- Power analysis has been added and its limited relevance for analyses of the pilot data described.
- A plan to accommodate potential participants with no mobile phone is now described.
- The number of formal courses and TA responsibilities have been reduced.
- Clarity is provided about the clinical trial aspects of the work, making clear that documentation required of clinical trials is managed by the sponsors, who lead the parent study.

Renewal:

Not applicable

Training in the Responsible Conduct of Research:

Acceptable

Comments on Format (Required):

- Web based certification courses, coursework, discussion with sponsors.

Comments on Subject Matter (Required):

- Ethics, research misconduct, human subjects research, informed consent, IRBs, intellectual property.

Comments on Faculty Participation (Required):

- Graduate course will be led by a faculty member; faculty sponsors will discuss ethics with applicant at regular meetings.

Comments on Duration (Required):

- Four-hour online course, portion of regular meetings with sponsors, semester-long graduate course.

Comments on Frequency (Required):

- Certification course every three years, graduate course on one occasion, biweekly meetings with sponsors.

Applications from Foreign Organizations:

Not Applicable

Select Agents:

Not Applicable (No Select Agents)

Resource Sharing Plans:

Not Applicable (No Relevant Resources)

Authentication of Key Biological and/or Chemical Resources:

Not Applicable (No Relevant Resources)

Budget and Period of Support:

Recommend as Requested

THE FOLLOWING SECTIONS WERE PREPARED BY THE SCIENTIFIC REVIEW OFFICER TO SUMMARIZE THE OUTCOME OF DISCUSSIONS OF THE REVIEW COMMITTEE, OR REVIEWERS' WRITTEN CRITIQUES, ON THE FOLLOWING ISSUES:

PROTECTION OF HUMAN SUBJECTS: ACCEPTABLE

INCLUSION OF WOMEN PLAN: ACCEPTABLE

INCLUSION OF MINORITIES PLAN: ACCEPTABLE

INCLUSION ACROSS THE LIFESPAN: ACCEPTABLE

COMMITTEE BUDGET RECOMMENDATIONS: The budget was recommended as requested.

Footnotes for 1 F31 DA058452-01A1; PI Name: Sibley, Adams Longstreet

+ Derived from the range of percentile values calculated for the study section that reviewed this application.

NIH has modified its policy regarding the receipt of resubmissions (amended applications).See Guide Notice NOT-OD-18-197 at https://grants.nih.gov/grants/guide/notice-files/NOT-OD-18-

197.html. The impact/priority score is calculated after discussion of an application by averaging the overall scores (1-9) given by all voting reviewers on the committee and multiplying by 10. The criterion scores are submitted prior to the meeting by the individual reviewers assigned to an application, and are not discussed specifically at the review meeting or calculated into the overall impact score. Some applications also receive a percentile ranking. For details on the review process, see http://grants.nih.gov/grants/peer_review_process.htm#scoring.

MEETING ROSTER

Center for Scientific Review Special Emphasis Panel
CENTER FOR SCIENTIFIC REVIEW
Fellowships: Clinical Care and Health Interventions

ZRG1 F16-L (20)
03/06/2023 - 03/07/2023

Notice of NIH Policy to All Applicants: Meeting rosters are provided for information purposes only. Applicant investigators and institutional officials must not communicate directly with study section members about an application before or after the review. Failure to observe this policy will create a serious breach of integrity in the peer review process, and may lead to actions outlined in NOT-OD-22-044 at https://grants.nih.gov/grants/guide/notice-files/NOT-OD-22-044.html, including removal of the application from immediate review.

CHAIRPERSON(S)

VANDERFORD, NATHAN LANE, PHD
ASSOCIATE PROFESSOR
DEPARTMENT OF TOXICOLOGY AND CANCER BIOLOGY
UNIVERSITY OF KENTUCKY
LEXINGTON, KY 40536

MEMBERS

ASLIBEKYAN, STELLA, PHD
ASSOCIATE PROFESSOR
DEPARTMENT OF EPIDEMIOLOGY
SCHOOL OF PUBLIC HEALTH
UNIVERSITY OF ALABAMA - BIRMINGHAM
BIRMINGHAM, AL 35294

BANDINI, LINDA G, PHD
PROFESSOR
DEPARTMENT OF PEDIATRICS
EUNICE KENNEDY SHRIVER CENTER
UNIVERSITY OF MASSACHUSETTS CHAN MEDICAL SCHOOL
WORCESTER, MA 01655

BERGE, JERICA M, PHD, MPH
PROFESSOR
DEPARTMENT OF FAMILY MEDICINE AND COMMUNITY HEALTH
UNIVERSITY OF MINNESOTA
MINNEAPOLIS, MN 55414

CARTER-BAWA, LISA, PHD
DIRECTOR
CANCER PREVENTION PRECISION CONTROL INSTITUTE
HACKENSACK MERIDIAN HEALTH
NUTLEY, NJ 07110

CARTUJANO, FRANCISCO, MD
DIRECTOR, COMMUNITY OUTREACH AND ENGAGEMENT
DEPARTMENT OF PUBLIC HEALTH SCIENCES
CENTER FOR COMMUNITY HEALTH AND PREVENTION
UNIVERSITY OF ROCHESTER MEDICAL CENTER
ROCHESTER, NY 14642

CHANG, MEI-WEI, PHD
ASSOCIATE PROFESSOR
COLLEGE OF NURSING
THE OHIO STATE UNIVERSITY
COLUMBUS, OH 43210

CHUANG, CYNTHIA H, MD
PROFESSOR
DIVISION OF GENERAL INTERNAL MEDICINE
MILTON S. HERSHEY MEDICAL CENTER
PENNSYLVANIA STATE UNIVERSITY
HERSHEY, PA 17033

COLWELL, GREGORY BRIAN, PHD
PROFESSOR
HEALTH PROMOTION AND COMMUNITY HEALTH SCIENCES
SCHOOL OF PUBLIC HEALTH
TEXAS A & M UNIVERSITY
COLLEGE STATION, TX 77843

CONROY, DAVID E., PHD
PROFESSOR
DEPARTMENT OF KINESIOLOGY AND HUMAN DEVELOPMENT &
FAMILY STUDIES
COLLEGE OF HEALTH AND HUMAN DEVELOPMENT
PENNSYLVANIA STATE UNIVERSITY
UNIVERSITY PARK, PA 16802

COOLEY-STRICKLAND, MICHELE R, PHD
PROJECT SCIENTIST
DEPARTMENT OF PSYCHIATRY AND BEHAVIORAL SCIENCES
CENTER FOR CULTURE AND HEALTH
UNIVERSITY OF CALIFORNIA
LOS ANGELES, CA 90024

COSTELLO, TRACY JENNIFER, PHD
DIRECTOR OF POSTDOCTORAL AFFAIRS AND GRAD STUDENT DEVELOPMENT
DEPARTMENT OF BIOSTATISTICS
UNIVERSITY OF SOUTH FLORIDA
TAMPA, FL 33612

DAVIS, ELIZABETH LENORE, PHD
ASSOCIATE PROFESSOR
DEPARTMENT OF PSYCHOLOGY
UNIVERSITY OF CALIFORNIA - RIVERSIDE
RIVERSIDE, CA 92521

DEW, MARY AMANDA, PHD
PROFESSOR
DEPARTMENT OF PSYCHOLOGY
UNIVERSITY OF PITTSBURGH
PITTSBURGH, PA 15213

DVORAK, ROBERT DANIEL, PHD
ASSOCIATE PROFESSOR
DEPARTMENT OF CLINICAL SCIENCES
THE UNIVERSITY OF CENTRAL FLORIDA
ORLANDO, FL 32816

EKAS, NAOMI VANESSA, PHD
ASSOCIATE PROFESSOR
DEPARTMENT OF PSYCHOLOGY
TEXAS CHRISTIAN UNIVERSITY
FORT WORTH, TX 76129

FOSTER, KENNETH R, PHD
PROFESSOR
DEPARTMENT OF BIOENGINEERING
SCHOOL OF ENGINEERING AND APPLIED SCIENCE
UNIVERSITY OF PENNSYLVANIA
PHILADELPHIA, PA 19104

FOTI, DANIEL JUSTIN, PHD
ASSOCIATE PROFESSOR
DEPARTMENT OF PSYCHOLOGICAL SCIENCES
COLLEGE OF HEALTH AND HUMAN SERVICES
PURDUE UNIVERSITY
WEST LAFAYETTE, IN 47907

FOX, AARON D, MD
ASSOCIATE PROFESSOR
DEPARTMENT OF MEDICINE
MONTEFIORE MEDICAL CENTER
ALBERT EINSTEIN COLLEGE OF MEDICINE
BRONX, NY 10467

HAAS, NIINA M.
VICE PRESIDENT
BRIGHTOUTCOME INC.
BUFFALO GROVE, IL 60089

HARVEY, IDETHIA SHEVON, MPH, DRPH
ASSOCIATE PROFESSOR
DEPARTMENT OF HEALTH SCIENCES
SCHOOL OF HEALTH PROFESSIONS
UNIVERSITY OF MISSOURI
COLUMBIA, MO 65211

HERON, KRISTIN E, PHD
ASSOCIATE PROFESSOR
DEPARTMENT OF PSYCHOLOGY
OLD DOMINION UNIVERSITY
NORFOLK, VA 23529

HOYLE, RICK, PHD
PROFESSOR
DEPARTMENT OF PSYCHOLOGY AND NEUROSCIENCE
DUKE UNIVERSITY
DURHAM, NC 27708

IRVIN, VERONICA L, MPH, PHD
ASSOCIATE PROFESSOR
COLLEGE OF PUBLIC HEALTH AND HUMAN SCIENCES
OREGON STATE UNIVERSITY
CORVALIS, OR 97331

KELLER, PEGGY S, PHD
ASSOCIATE PROFESSOR
DEPARTMENT OF PSYCHOLOGY
UNIVERSITY OF KENTUCKY
LEXINGTON, KY 40506

KENYA, SONJIA, EDD
ASSOCIATE PROFESSOR AND DIRECTOR
DIVISION OF GENERAL INTERNAL MEDICINE
MILLER SCHOOL OF MEDICINE
UNIVERSITY OF MIAMI
CORAL GABLES, FL 33146

KOUROS, CHRYSTYNA D, PHD
ASSOCIATE PROFESSOR
DEPARTMENT OF PSYCHOLOGY
SOUTHERN METHODIST UNIVERSITY
DALLAS, TX 75275

LEE, MIRYOUNG, MPH, PHD
ASSOCIATE PROFESSOR
DEPARTMENT OF EPIDEMIOLOGY, HUMAN GENETICS AND AND ENVIRONMENTAL SCIENCES
SCHOOL OF PUBLIC HEALTH
UNIVERSITY OF TEXAS HEALTH SCIENCE CENTER
BROWNSVILLE, TX 78520

MACNAMARA, ANNMARIE EILEEN, PHD
ASSOCIATE PROFESSOR
DEPARTMENT OF PSYCHOLOGICAL & BRAIN SCIENCES
TEXAS A & M INSTITUTE OF NEUROSCIENCE
TEXAS A & M UNIVERSITY
COLLEGE STATION, TX 77843

MARTIN, LAURA E, PHD
PROFESSOR
DEPARTMENT OF POPULATION HEALTH
UNIVERSITY OF KANSAS
KANSAS CITY, KS 66160

PELOSO, GINA MARIE, PHD
ASSOCIATE PROFESSOR
DEPARTMENT OF BIOSTATISTICS
BOSTON UNIVERSITY SCHOOL OF PUBLIC HEALTH
BOSTON, MA 02214

REDING, KERRYN W, PHD, MPH
ASSOCIATE PROFESSOR
SCHOOL OF NURSING
UNIVERSITY OF WASHINGTON
SEATTLE, WA 98195

ROUSSOS, STERGIOS, PHD, MPH
INTERIM EXECUTIVE DIRECTOR
HEALTH SCIENCES RESEARCH INSTITUTE
SCHOOL OF ENGINEERING
UNIVERSITY OF CALIFORNIA, MERCED
MERCED, CA 95340

SEGRE, ALBERTO MARIA, PHD
PROFESSOR
DEPARTMENT OF COMPUTER SCIENCE
THE UNIVERSITY OF IOWA
IOWA CITY, IA 52242

SEVCIK, ROSE A, PHD
PROFESSOR
DEPARTMENT OF PSYCHOLOGY
GEORGIA STATE UNIVERSITY
ATLANTA, GA 30303

SIMONSEN, SARA E, PHD
ASSOCIATE PROFESSOR
COLLEGE OF NURSING - MIDWIFERY
THE UNIVERSITY OF UTAH
SALT LAKE CITY, UT 84112

ST. HELEN, GIDEON, PHD
ASSOCIATE PROFESSOR
DIVISION OF CLINICAL PHARMACOLOGY
DEPARTMENT OF MEDICINE
UNIVERSITY OF CALIFORNIA
SAN FRANCISCO, CA 94143

TAYLOR, ROBERT JOSEPH, PHD
HAROLD JOHNSON ENDOWED PROFESSOR
SCHOOL OF SOCIAL WORK
INSTITUTE FOR SOCIAL RESEARCH
UNIVERSITY OF MICHIGAN
ANN ARBOR, MI 48109

TRAPL, ERIKA S, PHD
ASSOCIATE PROFESSOR
DEPARTMENT OF POPULATION AND
QUANTITATIVE HEALTH SCIENCES
CASE WESTERN RESERVE UNIVERSITY
CLEVELAND, OH 44106

TUBMAN, JONATHAN G, PHD
PROFESSOR
DEPARTMENT OF PSYCHOLOGY
AMERICAN UNIVERSITY
WASHINGTON, DC 20016

SCIENTIFIC REVIEW OFFICER

VO, HOA THI, PHD
SCIENTIFIC REVIEW OFFICER
CENTER FOR SCIENTIFIC REVIEW
NATIONAL INSTITUTES OF HEALTH
BETHESDA, MD 20892

Consultants are required to absent themselves from the room during the review of any application if their presence would constitute or appear to constitute a conflict of interest.
